# Bacterial Diversity in the Different Ecological Niches Related to the Yonghwasil Pond (Republic of Korea)

**DOI:** 10.3390/microorganisms12122547

**Published:** 2024-12-11

**Authors:** Myung Kyum Kim, Bong-Soon Lim, Chang Seok Lee, Sathiyaraj Srinivasan

**Affiliations:** Department of Bio and Environmental Technology, College of Natural Science, Seoul Women’s University, Seoul 01797, Republic of Korea

**Keywords:** Yonghwasil pond, freshwater ecosystems, bacterial diversity, National Institute of Ecology

## Abstract

The bacteriome profile was studied in freshwater ecosystems within the Yonghwasil pond, situated at the National Institute of Ecology, Seocheon-gun, Chungcheongnam-do, central western Korea. Six samples from water, mud, and soil niches were assessed, specifically from lake water, bottom mud (sediment), and root-soil samples of Bulrush, wild rice, Reed, and Korean Willow. Notably, the phylum *Actinobacteria* exhibited an upward trend moving from water to mud to soil samples, whereas *Chloroflexi* showed a contrasting decrease. Across the board, *Proteobacteria* emerged as the reigning phylum, and subsequent dominance was attributed to *Firmicutes* and *Actinobacteria*. The water samples were characterized by an enriched presence of *Cyanobacteria* and *Bacteroidetes*, whereas the mud samples distinctly housed a higher concentration of *Chloroflexi*. Assessing biodiversity through OTU and ACE indices revealed a subdued species richness in the water samples. On the contrary, mud samples stood out with the highest OTU and ACE metrics, signifying a microbially diverse habitat. Bulrush, wild rice, Reed, and Willow samples showed intermediate microbial diversity. The Shannon index further corroborated the pronounced microbial diversity in mud and Bulrush habitats with others. This research elucidates the microbial intricacies across different habitats within Yonghwasil Pond, emphasizing the pivotal role of environmental matrices in shaping bacterial communities.

## 1. Introduction

Freshwater habitats, which constitute only a small portion of the overall water volume on Earth, hold great importance due to their support of a wide range of species and their fundamental role in promoting human well-being [1,2,3]. Lakes and ponds, commonly called “lentic jewels” within our landscapes, represent distinctive habitats that provide various ecological, economic, and cultural advantages [4]. Lakes and ponds, characterized by their tranquil waters and stratified layers, serve as significant hubs of biodiversity. The freshwater ecosystems serve as crucial repositories of genetic diversity, hosting a substantial number of endemic species [5,6,7]. In addition, biodiversity in lakes and ponds offers a wide range of vital ecosystem services. Wetlands serve as effective natural mechanisms for water purification, effectively removing contaminants and guaranteeing the availability of potable water [1,8,9]. The sediments significantly influence the biogeochemical cycling process, impacting the movement of nutrients and elements throughout ecosystems.

Furthermore, it is imperative to acknowledge the significance of these aquatic ecosystems in the context of agriculture, as they play a crucial role in acting as reservoirs for irrigation purposes and ensuring food security for extensive populations [1,10,11]. From a hydrological perspective, lakes and ponds serve a crucial function in regulating water flow, functioning as important buffers that mitigate the impacts of floods and droughts. These entities play a crucial role in replenishing groundwater, impacting nearby and broader water levels [12,13,14]. In addition, it is essential to consider the thermal dynamics of more giant lakes as they can affect local climatic conditions, influencing temperature and precipitation patterns in the surrounding area [1,15,16].

The preservation of these freshwater environments is currently being jeopardized. The consequences of such degradation are diverse, affecting not just the species they contain but also the wide range of ecosystem services they offer. Given these issues, it is crucial to comprehend and underscore the significance of lakes and ponds in the broader framework of freshwater ecosystems [17]. The complex network of organisms in freshwater habitats, such as lakes and ponds, relies on a varied and ever-changing microbiome. The microbial communities found in these aquatic habitats, primarily consisting of bacteria, significantly impact the ecological equilibrium, nutrient cycling, and overall well-being of these environments [18]. The microbiome in freshwater habitats holds great significance due to its pivotal role in driving critical biogeochemical processes, making it an indispensable force that should not be underestimated. The presence of nearby terrestrial ecosystems, namely vegetation, significantly influences the structure and functionality of microbial communities within lakes and ponds [19]. The dynamic relationship between terrestrial and aquatic ecosystems serves as evidence of the inherent interconnection of the natural world, wherein even minute microbes are subject to the effect of the more significant macroscopic ecosystem [20].

Plants and trees contribute to the discharge of organic matter into water bodies through the action of their root systems and the deposition of leaf litter. These substances function as a plentiful reservoir of nutrients for microbial populations, facilitating their proliferation and metabolic processes. The symbiotic link between the microbiota and plants facilitates an ongoing process of nutrient exchange, enhancing the ecosystem’s overall well-being and efficiency. In addition, vegetation, specifically plants and trees, in the vicinity of lakes and ponds establishes a protective barrier, thereby mitigating the entry of contaminants and particulate matter into the water bodies [21]. The microbial communities play a crucial role in water purification by decomposing pollutants and absorbing excessive nutrients, hence mitigating the occurrence of eutrophication and algal blooms [21]. The microorganisms’ metabolic adaptability enables them to engage in diverse ecological roles, encompassing carbon sequestration and nitrogen fixation [22]. The presence of neighboring plants and trees significantly impacts the diversity and activity of the bacterial population, hence contributing to the development of a more resilient and stronger ecosystem. Human activities, such as deforestation, urbanization, and pollution, can potentially disturb the delicate balance of ecosystems, resulting in alterations in the composition and functioning of microbial communities [22,23]. The depletion of vegetation, specifically plant and tree cover, results in the deprivation of vital organic inputs to the microbiome, compromising the symbiotic association that upholds the ecosystem. The overall condition and sustainability of lakes and ponds are contingent upon the welfare of their microbial populations and the vegetation, including plants and trees, which provide them with sustenance. As the investigation into the intricacies of these relationships progresses, it becomes evident that preserving the integrity of terrestrial and aquatic ecosystems is crucial for the long-term viability of our planet [2].

Recently, there has been a notable increase in the focus on the complex interconnections between aquatic ecosystems and the microorganisms inside them. This emphasis has shed light on the substantial impact that these microorganisms have on the overall health and functioning of these ecosystems [24]. In Korea, while the aquatic zone has remained well-conserved, the riparian zones in most lakes have undergone significant disappearance. This phenomenon primarily stems from the widespread conversion of wetlands into rice paddies, a staple food crop cultivated in aquatic environments. Given Korea’s minimal exposure to glacial activity, natural lakes are concentrated along the east coast, with the remainder being artificial reservoirs. Surrounding these lakes are expanses of rice fields dedicated to rice cultivation, resulting in the continuous management of riparian areas [25]. Consequently, these areas seldom exhibit full riparian vegetation, except in designated conservation zones, due to the shading effects caused by the growth of riparian vegetation, which can impede rice growth.

Yonghwasil Pond, located within the National Institute of Ecology, was originally constructed as an agricultural reservoir with a rectangular shape and steep slopes. The pond experienced severe eutrophication due to wastewater from nearby livestock complexes and agricultural lands, resulting in a simplistic vegetation structure, predominantly *Zizania latifolia*. *Trapa japonica* covered the deeper waters, forming a dense surface mat—a common feature in eutrophic ponds. To restore the ecological balance, substantial modifications were implemented. The pond was reshaped into an elliptical form, and its depth profile was diversified, with the center reaching up to 2.5 m and gradually becoming shallower toward the edges. Steep slopes were contoured to enhance connectivity between aquatic and riparian zones. Following these changes, an adaptive management approach was adopted, introducing riparian and emergent plants to stabilize the ecosystem, while allowing the natural establishment of other plant species. Over a decade, these efforts have resulted in a vegetation profile resembling that of a natural lake, supporting various plant forms like free-floating, submerged, emergent, and wetland species.

The primary aim of this work is to study the novel composition and diversity of bacterial communities in Yonghwasil Pond, focusing on their heterogeneity across different ecological niches. To achieve this, six samples were collected, from both aquatic and terrestrial interfaces, including lake water, bottom mud, and rhizosphere soil from four dominant vegetation types: Bulrush *(Typha orientalis*), wild rice *(Zizania latifolia*), Reed (*Phragmites australis*), and Korean Willow (*Salix pierotii*). This study highlights the importance of microbial diversity in freshwater ecosystems and its role in nutrient cycling, water purification, and ecosystem balance. The findings have practical applications in habitat restoration, water quality management, and sustainable ecosystem practices. The diverse vegetation plays a crucial role in shaping microbial communities through root exudates and organic debris. By analyzing these bacterial communities, the study provides comprehensive insights into the ecological significance and interactions of microbes within this restored freshwater ecosystem.

## 2. Materials and Methods

### 2.1. The Study Site

**Figure 1:**
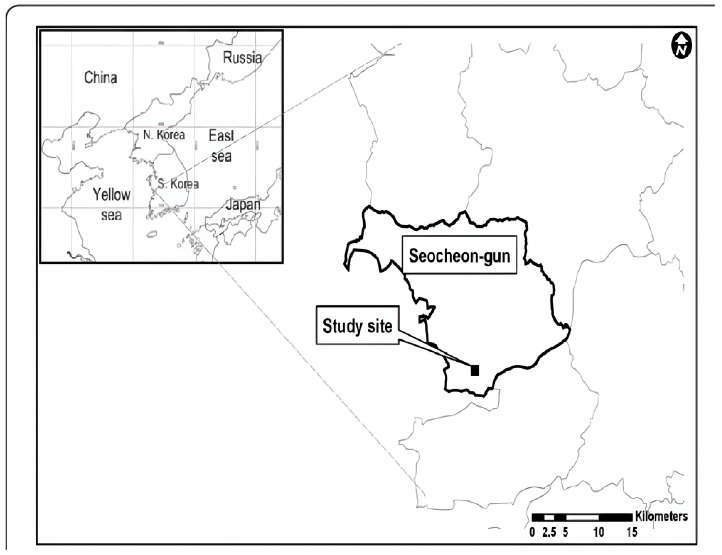
A map showing the geographic location of the National Institute of Ecology, which is located in Seocheon, Chungcheongnam-do, South Korea.

The study was conducted at Yonghwasil Pond, located within the National Institute of Ecology in Seocheon-gun, Chungcheongnam-do, central western Korea (N 36°2.508′ E 126°43.110′) (Figure 1). Initially an agricultural reservoir, the pond underwent ecological restoration to address eutrophication and reshape its structure. The restoration expanded the pond’s size, diversified its depth profile, and enhanced connectivity between aquatic and riparian zones. These efforts transformed Yonghwasil Pond into a dynamic freshwater ecosystem, supporting diverse vegetation and microbial communities. The site now features various water depths and plant zones, providing a suitable habitat for studying bacterial diversity and interactions across multiple ecological niches. The pond’s diverse habitats, including open water, mudflats, and vegetated areas, offer a range of microenvironments for bacterial colonization. This variety allows for comprehensive sampling to investigate the ecological roles and spatial distribution of bacterial communities.

### 2.2. Sample Collection

The sample collection was carried out at Yonghwasil Pond, a prominent aquatic habitat inside the National Institute of Ecology grounds in Seocheon-gun, Chungcheongnam-do. Yonghwasil Pond was selected for in-depth investigation based on its recognition as a highly abundant reservoir of diverse ecological specimens. The study was conducted in three primary niches within the pond ecosystem: water, mud, and soil. Six unique samples were found and selected from these niches, taking into consideration their ecological significance and prevalence. The samples collected for analysis encompassed various environmental components, namely lake water (L1-W), bottom sediment (L1-M), Bulrush (L1-T), wild rice (L1-Z), Reed (L1-R), and Korean Willow (L1-B) (a schematic diagram showing the samples is included in the Supplementary Figure S1).

Four liters of surface water was collected separately from each of the four distinct locations within the pond using sterile containers, ensuring representative sampling for each site. This methodology ensured a thorough and inclusive depiction of the water composition within the pond. For DNA isolation, samples were filtrated on a 0.2 μm filter paper [26]. Four mud samples were collected from different regions within the pond to provide a comprehensive representation of the sediment profile. The samples were specifically taken from the lower strata of the pond at a depth of approximately 2 m. This depth was selected based on its ability to offer a combination of stable organic material and microorganisms that inhabit such ecological niches. Close to the lake, along its periphery, the proliferation of various plant species including Bulrush, wild rice, and Reed was sighted. The roots of these plants are deeply embedded in muddy soil, forming a key part of the pond’s ecosystem. Soil samples were collected near the roots to capture their composition and associated microorganisms. Similarly, soil was sampled near the roots of the Korean Willow, a prominent tree thriving at the pond’s periphery under suitable conditions.

Regarding quantity, it is worth noting that the pond water and mud samples were obtained through four separate collections. In contrast, the four plant-based samples (Bulrush, wild rice, Reed, and Korean Willow) were gathered in duplicate. This measure ensured the maintenance of uniformity within our dataset. Each sample was carefully and securely enclosed into transparent ziplock bags, guaranteeing the prevention of any potential external contamination. Subsequently, the bags were subjected to a controlled temperature of 4 °C to establish a consistent and controlled setting until they could be transported to the laboratory for subsequent analysis. In the laboratory setting, the materials were preserved at a significantly lower temperature of −80 °C to mitigate degradation and facilitate their suitability for subsequent experiments.

### 2.3. The Soil Chemical Properties

For the comprehensive study, we analyzed soil samples’ various physical and chemical properties. These properties encompassed pH, TN (Total Nitrogen; %), TP (Total Phosphate, mg/kg), TOC (Total Organic Carbon, %), NH^4+^ (Ammonium mg/kg), and NO^3−^ (nitrate mg/kg). This analysis was conducted at the National Instrumentation Center for Environmental Management (NICEM), Seoul, Republic of Korea. The methodologies for this analysis strictly adhered to the standardized protocols set forth by the Soil Science Society of America. The pH level of the soil samples was determined by employing a combined pH electrode. For this, a suspension was prepared by mixing samples with tap water in a 1:2 ratio, following the methodology delineated by Kalra [27]. One g of sample was digested using sulfuric acid to quantify the Total Nitrogen (TN). Therefore, this process was carried out using Se, CuSO_4_, and K_2_SO_4_ as catalysts. The final TN content in the digested sample was then determined using the conventional Kjeldahl distillation method, as described in [28]. The phosphorus content of the soil samples was gauged using the method established by Bray and Kurtz [29]. An autoanalyzer was employed for this purpose, and the analysis was conducted using 3 g of samples. The samples’ Total Organic Carbon (TOC) content was ascertained through an oxidation process involving 1 N potassium dichromate within an acidic environment. This method was followed based on pioneering works of Rowell and Florence [30], further elaborated by Rowell [31]. The concentration of NH^4+^ (mg/kg) in the soil was measured using the dithionite–citrate system buffered with sodium bicarbonate, as proposed by Mehra and Jackson (1958) [32]. Lastly, the quantification of NO^3−^ (mg/kg) was carried out using the colorimetric method, as described by Arnold et al. (1998) [14]. Adhering to these rigorous methodologies, this study aimed to provide a holistic and accurate assessment of the soil’s chemical properties.

### 2.4. DNA Isolation

For total DNA isolation, the filtrate collected from the 0.2 μm filter paper was used for water samples, while mud and soil samples were dried before DNA extraction. A total of 0.5 g of each sample was processed using the PowerSoil DNA Extraction Kit (MoBio Laboratories, Carlsbad, CA, USA), following the manufacturer’s protocol. The concentration and purity of the extracted DNA were determined using spectrometric absorbance measurements at 230–280 nm with a NanoDrop ND-1000 Spectrophotometer (NanoDrop Technologies, Wilmington, DE, USA) and verified with a Qubit 3 Fluorometer (Thermo Fisher Scientific, Waltham, MA, USA). The DNA samples were stored at −80 °C until further polymerase chain reactions (PCR).

### 2.5. PCR Amplification and Sequencing

The PCR amplification on the hypervariable region V3-V4 of the bacterial 16S rRNA was performed [33,34] using primers 341F (CCT ACG GGN GGC WGC AG) and 805R (GAC TAC HVG GGT ATC TAA TCC). The PCR conditions were established in the following conditions: 98 °C for 30 s, denaturation at 98 °C for 10 s, an annealing phase at 55 °C for 30 s, an elongation step at 72 °C for 30 s, and a final extension step at a temperature of 72 °C for 5 min.

Following the polymerase chain reaction (PCR) amplification, the sequencing process was conducted utilizing the Illumina platform (San Diego, CA, USA). The library creation process for sequencing was methodically executed by the established protocol supplied by Illumina, which may be obtained via the following link (https://sapac.illumina.com/techniques/sequencing/ngs-library-prep.html; accessed on 12 December 2022). The library was created using the Nextera XT kit (Illumina, San Diego, CA, USA) following the manufacturer’s instructions. A quantitative assessment was conducted on the individual amplicons of the V3-V4 region in each reaction combination, and the Illumina sequence adaptor was integrated. The emulsion PCR technique utilized the Nextera XT Index kit to enable the production of amplicon libraries. Subsequently, a polymerase chain reaction (PCR) cleanup procedure was implemented. The MiSeq libraries were quantified, followed by a 300-nucleotide paired-end multiplex sequencing on the Illumina MiSeq sequencer. This sequencing approach was used to ensure thorough and precise sequencing outcomes.

### 2.6. Sequencing Data Analyses

The demultiplexed paired-end (PE) raw reads generated by the Illumina MiSeq platform were evaluated for quality using FastQC (v0.12.1) [35] and then subjected to trimming using Trimmomatic (v0.39) [36]. To improve the quality of reads (Phred score Q > 30) and facilitate taxonomy categorization, the paired reads from each sample were combined using VSEARCH (v 2.23.0). The processed reads were subsequently loaded into QIIME2 (version 2023.5) for comprehensive analysis. The execution of quality control methods, such as filtering, trimming, and denoising, was performed using the q2-dada2 tool (https://github.com/qiime2/q2-dada2; accessed on 12 December 2022) [37]. The DADA2 algorithm was utilized to perform read dereplication, estimate error rates and infer sample sequence variants. The amplicon sequence variant (ASV) table was constructed, and chimeras were eliminated using the DADA2 method. The taxonomic and species assignments were facilitated using the DADA2 algorithm, concerning the SILVA v138.1 database [38], which was obtained on 15 August 2022. Various metrics were calculated to assess the richness and diversity of bacteria. These metrics included the Observed feature, Shannon, Chao, ACE indices, and Rarefaction curves. Additionally, Weighted and Unweighted Unifrac distance matrices were computed, and PCoA plots were generated. The statistical analyses of the obtained data were performed using GraphPad Prism 8.

## 3. Results

### 3.1. Soil Physico-Chemical Properties

The physico-chemical complexities of soil and water samples were comprehensively investigated with painstaking attention to detail. The pH of the lake water (L1-W) was measured to be slightly acidic at 4.9, and it exhibited significant quantities of Total Nitrogen (TN) and Total Organic Carbon (TOC) at 0.96% and 8.2%, respectively. In sharp juxtaposition, the sediment located at the lowermost portion of the lake (referred to as L1-M) displayed a pH level of 5.9, which was notably higher than expected. Additionally, this sediment demonstrated an exceptionally high Total Phosphorus (TP) content, measuring at 1091.29 mg/kg. These findings suggest that the sediment in this area is abundant in nutrients. The pH values of the rhizosphere mud samples obtained from several vegetation types, including Bulrush (L1-T), wild rice (L1-Z), and Reed (L1-R), ranged from 5.6 to 5.7. Out of the options provided, the Reed sample had the most notable characteristic, as it displayed the greatest Total Organic Carbon (TOC) content, measured at 10.68%. The soil sample obtained from Willow (L1-B) exhibited a comparable pH pattern 5.6. However, it is interesting that this sample displayed a significantly elevated nitrate concentration of 21.32 mg/kg (Table 1).

### 3.2. Bacterial Community Abundance and Composition

**Figure 2:**
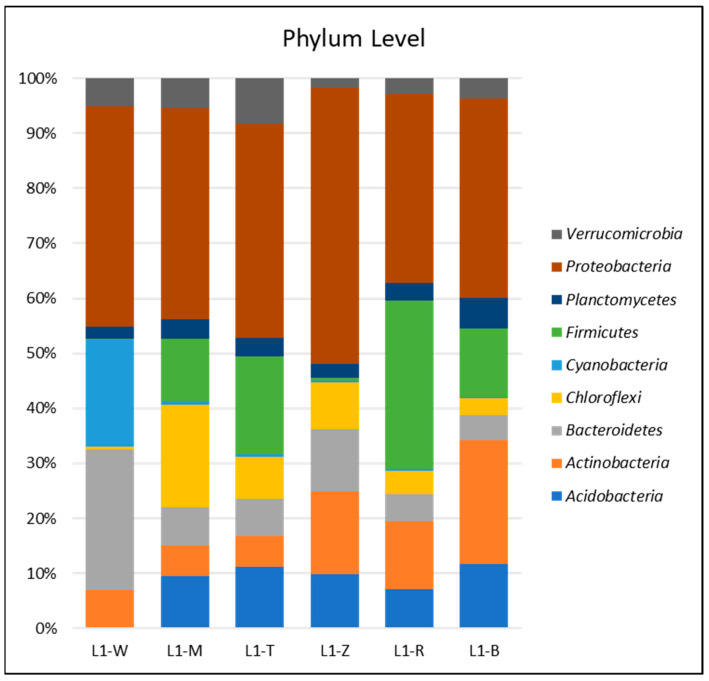
Phylum-level bacterial relative abundance. The bar chart represents the relative abundance of different bacterial phyla identified in the samples. The accompanying legend indicates that each color corresponds to a distinct bacterial phylum. The samples include water (L1-W), mud (L1-M), and rhizosphere soils from four dominant vegetation types: Bulrush (L1-T), wild rice (L1-Z), Reed (L1-R), and Korean Willow (L1-B).

The bacterial communities’ composition and abundance within pond habitats provide valuable insights into the influence of surrounding vegetation within the complex interconnections of these ecosystems. When examining the quantity of bacteria at the phylum level (Figure 2), trees and plants provide a distinct representation. The water samples (L1-W) were found to be composed of *Bacteroidetes* (44.87%) and *Cyanobacteria* (34.15%). In sharp contrast, the mud samples labeled L1-M displayed a notable prevalence of *Acidobacteria* (15.00%) and *Chloroflexi* (29.24%). It is worth mentioning that the samples related to *Typha orientalis* (commonly known as Bulrush, designated as L1-T) and *Zizania latifolia* (also known as wild rice, designated as L1-Z) exhibited an equitable distribution throughout various phylum. The Bulrush samples exhibited a prevalence of *Acidobacteria* (18.26%) and *Actinobacteria* (8.89%), but the wild rice samples displayed a higher proportion of *Acidobacteria* (16.52%) and *Actinobacteria* (25.25%).

**Figure 3:**
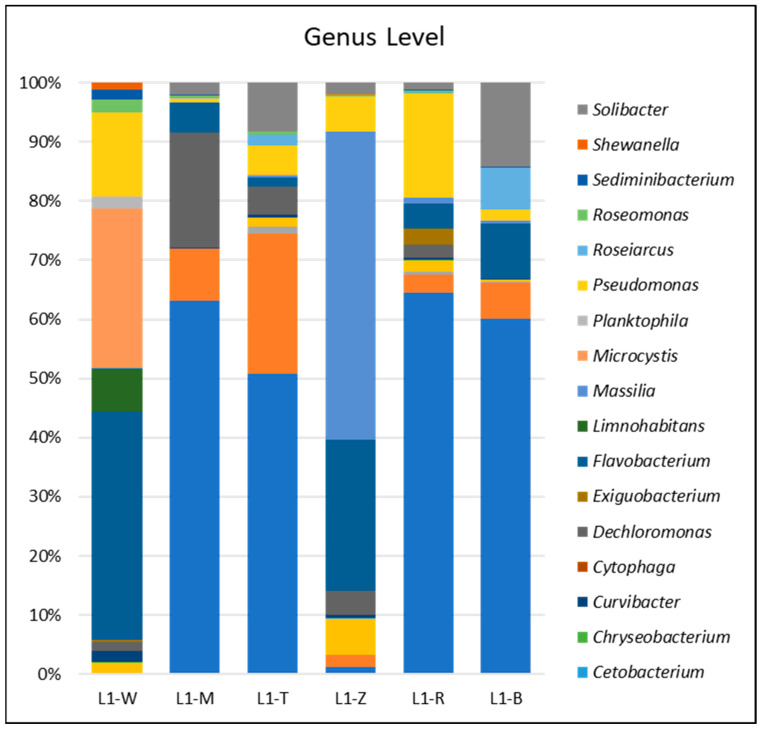
Genus-level bacterial relative abundance. The bar chart illustrates the relative abundance of bacterial genera across the collected samples. The analyzed samples are water (L1-W), mud (L1-M), and rhizosphere soils from Bulrush (L1-T), wild rice (L1-Z), Reed (L1-R), and Korean Willow (L1-B). Each color represents a distinct bacterial genus.

At a genus level, the distribution of genus further emphasizes the importance of plants in influencing the diversity of bacteria (Figure 3). The mud samples (L1-M) exhibited predominant colonization by *Clostridium* (16.23%) and *Geobacter* (14.08%). In the study, conducted by Trivedi et al. [39], it was observed that certain plants can support distinct bacterial communities. Similarly, our analysis of Bulrush samples (L1-T) revealed a substantial presence of *Clostridium* (18.51%) and *Geobacter* (52.84%), which aligns with the results previously reported. The Reed samples (L1-R) exhibited a significant presence of *Clostridium* (42.30%), indicating its probable function as an accumulation for this specific species.

This bar chart showcases the relative abundance of various bacterial genera identified within the analyzed samples. Each color corresponds to a unique bacterial genus, delineated in the accompanying legend. Investigations into the cumulative number of species across varying ecological niches from mud, aquatic zones, and vegetation to riparian vegetation reveal a compelling narrative on the foundational role of ecological diversity as a precursor to species diversity within the Yonghwasil pond ecosystem. The gradient observed from bottom mud to aquatic environments, extending through areas dominated by Bulrush, wild rice, and Reed, reaching the zones with dense riparian vegetation, underscores a significant increase in species richness.

### 3.3. Correlation of Soil Properties and the Bacterial Community

The bacterial community structure in pond habitats is significantly influenced by the soil’s phytochemical properties, particularly those affected by the surrounding flora. Distinct trends can be observed in the dataset, depending on the types of samples analyzed. These samples encompass a range of sources, including direct water samples and samples connected with particular plant species such as Bulrush (*Typha orientalis*), wild rice (*Zizania latifolia*), Reed (*Phragmites communis*), and Willow (*Salix pierotii*).

**Figure 4:**
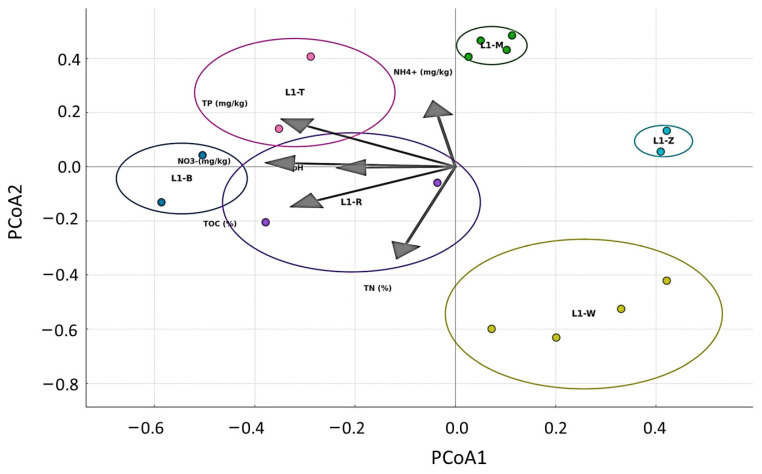
PCoA plot showing microbial community structures across different sample types. Clustering patterns reflect variations among water (L1-W), mud (L1-M), and rhizosphere soil samples from Bulrush (L1-T), wild rice (L1-Z), Reed (L1-R), and Korean Willow (L1-B). Arrows indicate environmental variables influencing microbial distribution.

Principal Coordinate Analysis (PCoA) of microbiome data revealed a substantial differentiation in microbial community compositions across various sample types. The samples exhibited a strong clustering pattern depending on their environmental context, wherein samples from similar habitats, such as mud, tended to group together closer to the ordination space. This finding suggests the habitat’s significant impact on the microbial community’s structure (Figure 4).

In addition, the ordination lines depicting different chemical variables offer valuable insights into the environmental conditions contributing to the observed variations in microbial communities. For example, the variables Total Organic Carbon (TOC) and Ammonium (NH^4+^) displayed prominent vectors in the Principal Coordinate Analysis (PCoA) space, indicating that they could play a significant role in the observed microbial differentiation. The directional vectors of these entities demonstrate potential associations with the spatial arrangement of specific microbial populations. It is important to note that the microbial community architectures of the soil and water samples exhibited discernible differences, as seen by their separation from the mud samples in the PCoA plot. The differentiation highlights the diverse microbial communities linked to these specific environments. The investigation of chemical gradients, such as pH and Total Nitrogen (TN), contributes to a more nuanced comprehension of the microbial preferences particular to different habitats.

**Figure 5:**
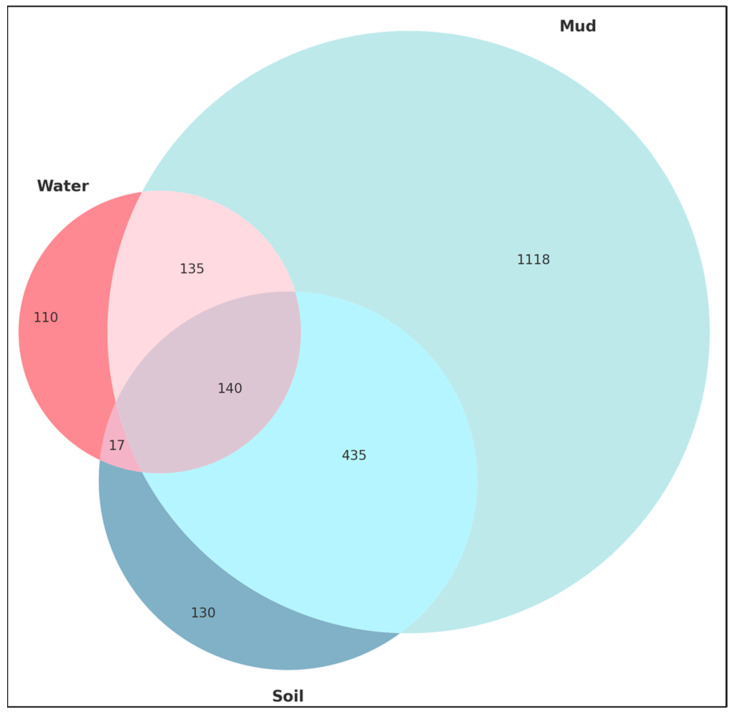
Venn diagram of soil microbiome across different sample groups. The Venn diagram illustrates the shared and unique bacterial genera across water (L1-W), mud (L1-M), and rhizosphere soil samples from Bulrush (L1-T), wild rice (L1-Z), Reed (L1-R), and Korean Willow (L1-B). Overlapping regions represent genera common to multiple sample types, while non-overlapping sections highlight unique bacterial genera specific to each sample type.

The Venn diagram clearly represents the overlap and distinctions in bacterial genera presence among the water, mud, and soil sample groups (Figure 5). Notable intersections indicate shared genera, suggesting potential common ecological or environmental factors influencing their occurrence. Unique segments within each circle highlight the distinct bacterial communities characterizing each environmental condition. The equal circle areas ensure a balanced comparison across the three sample groups. In conclusion, the composition of microbial communities is influenced by both habitat type and specific chemical characteristics, as demonstrated using PCoA and Venn diagram visualization. These insights are crucial in comprehending microbial ecology and the underlying processes influencing community assembly across diverse settings.

The study underscores the unique attributes of microorganism communities in different environmental habitats within Yonghwasil Pond. These findings reveal the intricate interplay of life forms adapted to different environments, including mud, aquatic zones, plants, and riparian vegetation. The mud samples show a strong abundance of *Acidobacteria* and *Chloroflexi*, indicating a community well suited to thrive in nutrient-rich, anaerobic environments. Conversely, the aquatic zone is primarily inhabited by *Proteobacteria* and *Cyanobacteria*, demonstrating a diverse and adaptable microbial community that can thrive in varying water conditions. The presence of microorganisms in the vicinity of aquatic plants, particularly Bulrush and wild rice, exhibits a notable level of diversity. This diversity includes *Actinobacteria*, which are known to have a significant impact on the breakdown of organic matter and the cycling of nutrients. These microbes contribute to the improvement of plant health and the overall quality of water. The riparian vegetation zones contain a distinct combination of microbial life, with a greater presence of *Bacteroidetes* and *Firmicutes*. This suggests a transitional area where aquatic and terrestrial microbial communities come together, emphasizing the important role of riparian zones in connecting water bodies with terrestrial ecosystems. The results highlight the ecological importance of preserving a variety of habitats in freshwater environments, as each niche sustains a distinct microbial community that plays a crucial role in the overall biodiversity and ecological functioning of the pond.

## 4. Discussion

This study underscores the critical role of niche-specific environmental conditions in shaping bacterial communities within freshwater ecosystems like Yonghwasil Pond. These microbial communities, with their complex and dynamic nature, are essential to the ecosystem’s biogeochemical processes, trophic interactions, and overall ecological health. The importance of biodiversity extends beyond academic interest; it is fundamental to the stability and resilience of the environment [40,41]. Biodiversity reflects the heterogeneity of habitats [42,43,44] and indicates an ecosystem’s integrity, where a highly diverse habitat often signals a balanced, well-functioning environment [45,46].

These results align with the prevailing scientific opinion that emphasizes the pivotal contribution of trees and plants in fostering advantageous bacterial communities within aquatic ecosystems [20]. The influence exerted by the rhizosphere of these trees and plants on the bacterial community is clearly apparent. As an illustration, the rhizosphere sediment linked to specific plants exhibited a notable proliferation of bacterial phyla, suggesting the presence of a more diverse microbial community. The observed diversification can be attributed to the intricate phytochemical substances emitted by the plants, which foster a favorable habitat for a broader array of bacterial species. These discoveries provide support for previous research that suggested certain types of vegetation significantly impact the diversity of bacteria in their surrounding environment, thereby affecting the general health of ecosystems [2,30,33,47]. In contrast, samples not influenced by these trees and plants, such as strait water and mud samples, had a distinct bacterial community structure. The lack of specific bacterial phyla observed in these samples indicates that the absence of phytochemical inputs from the plants could potentially disrupt the microbial balance of the pond, hence compromising bacterial diversity [36].

The evidence presented in our study supports the perspective presented [20,47,48], which highlights the interdependent interaction between flora and bacterial communities in pond ecosystems. The existence of a variety of plant life, including trees and aquatic plants, plays a crucial role in promoting and sustaining a harmonious bacterial community, which is fundamental to the well-being and robustness of pond ecosystems [47]. In brief, the interaction between the phytochemical characteristics of soil and its bacterial community is intricately regulated by the presence of particular trees and plants. These results emphasize the significance of conserving indigenous flora in the vicinity of pond ecosystems to sustain their microbial equilibrium and overall well-being [47].

The restoration of Yonghwasil Pond has led to increased biodiversity, primarily driven by morphological changes, such as transforming its shape from rectangular to oval, reducing steep slopes, increasing water depth, and improving water quality. These changes have supported a more diverse range of habitats, introducing riparian and emergent vegetation zones. Specifically, water depth plays a pivotal role in the establishment of vegetation and the assembly of microbial communities [49,50]. Diverse depth zones enhance species richness and promote ecological heterogeneity [51,52]. Furthermore, studies by [53,54,55] highlight the strong relationship between vegetation diversity and the heterogeneity of water depth. As anthropogenic pressures on freshwater systems continue to rise, understanding the drivers of microbial and ecological diversity, as demonstrated in this study, is crucial for guiding effective conservation and restoration strategies.

### 4.1. Proteobacteria: The Ecological Workhorse

The ubiquity of *Proteobacteria* in freshwater ecosystems, as observed in our study, is a testament to their ecological versatility. In addition to their metabolic flexibility, the ecological success of *Proteobacteria* can be attributed to their prompt reaction to changes in nutrition availability, their capacity to establish symbiotic associations with eukaryotic hosts, and their inclination towards horizontal gene transfer, which enhances their adaptability [47,56]. Multiple studies have consistently identified *Proteobacteria* as a prominent constituent in many freshwater environments [20,56,57]. The vital importance of these organisms in maintaining ecological balance, particularly in nutrient cycling, is underscored by their dominance in various niches within the pond. Research has indicated that many classes within the *Proteobacteria* phylum exhibit diverse functions, encompassing sulfur metabolism and carbon cycle [56,58]. The prevalence of their dominance may also suggest particular patterns of organic matter dynamics occurring inside the pond. For example, elevated levels of labile organic matter may promote the proliferation of specific proteobacterial groups that are recognized for their accelerated rates of growth [59].

### 4.2. Actinobacteria and Chloroflexi: Indicators of Environmental Gradients

*Actinobacteria*, renowned for their resilience in terrestrial environments, find a pronounced presence as we move from aqueous to semi-solid to solid matrices. Their ability to produce mycelium-like structures might give them a strategic advantage in colonizing and extracting nutrients from solid substrates [60,61]. *Actinobacteria’s* rise in concentration can be attributed to their knack for producing many secondary metabolites, including antibiotics [62,63,64]. Their increasing dominance from water to mud to soil could also hint at a gradient of organic complexity, with soil and mud niches potentially harboring more complex organic compounds. These metabolites could provide them with a competitive edge in the nutrient-rich mud and soil environments, warding off potential competitors. The differential distribution of *Actinobacteria* and *Chloroflexi* suggests distinct environmental gradients within the pond. *Chloroflexi’s* decline across the gradient is evocative of their specific habitat preferences. *Chloroflexi* might be more sensitive to oxygen gradients, with specific groups favoring more anoxic conditions [65]. *Chloroflexi* distribution could thus provide insights into the redox conditions of different niches. Their primary role in carbon cycling, especially in anaerobic conditions, might make them more suited for water habitats where oxygen penetration is limited [61,66].

### 4.3. Cyanobacteria and Bacteroidetes in Water Samples: More than Just Photosynthesis

While *Cyanobacteria’s* photosynthetic capabilities are well-documented, their role in the microbial food web is equally crucial. They can be primary producers, providing essential organic substrates for heterotrophic bacteria. Furthermore, certain cyanobacterial groups can fix atmospheric nitrogen, supplementing the nitrogen pool in nutrient-limited waters [67,68]. However, their dominance could also be a double-edged sword. Under eutrophic conditions, blooms of certain cyanobacterial species can produce toxins detrimental to aquatic life [69]. Their presence could indicate the nutrient status of the Yonghwasil pond, with potential implications for water quality and trophic dynamics. Concurrently, the prominence of *Bacteroidetes* speaks volumes about the organic material dynamics in the lake water. Renowned for their capability to degrade high molecular weight organic compounds, their abundance suggests a continuous flux of such compounds, possibly from decaying aquatic vegetation or allochthonous sources [5,70].

### 4.4. Sediments: The Hidden Reservoir of Biodiversity

Sediments often hold the key to understanding aquatic ecosystem dynamics. The microbial diversity in mud samples suggests many mutualistic and antagonistic interactions. Their role as sinks for organic and inorganic matter makes them hotspots for biogeochemical transformations [70,71]. The heightened microbial diversity might also reflect the mud’s varied particle sizes and organic matter composition, each fostering unique microhabitats. These interactions can influence nutrient cycling, organic matter decomposition, and even pollutant degradation [72]. Significantly, nitrates were undetectable in multiple mud samples, which aligns with the conclusions drawn by Mulder and Schmidt [73]. The study highlights the significance of specific wetland plants in nitrate absorption. This absence of nitrates suggests active nitrate assimilation processes, with the microbial community playing a critical role in nutrient cycling. Exploring the functional roles of these sediment bacteria further could unravel the hidden processes underpinning the pond’s ecology, particularly in relation to nitrogen cycling and its broader ecological impact.

### 4.5. Aquatic Plants: Mediators of Microbial Interactions

The intermediate microbial diversity observed in root mud samples from Bulrush, wild rice, Reed, and Willow offers a fascinating narrative. The role of aquatic plants extends beyond photosynthesis. Their roots release a plethora of organic compounds, collectively termed root exudates. These exudates can attract specific bacterial groups, influencing the immediate rhizosphere’s microbial community [74]. Furthermore, decaying plant matter can provide substrates for detritivorous bacteria, influencing sediment microbial dynamics. Bacterial communities in these niches likely benefit from a dual nutrient source: the settling organic matter from the water column and the root exudates from the aquatic plants [75]. This confluence of resources might lead to intermediate diversity, striking a balance between the water and mud extremes.

### 4.6. Potential Anthropogenic Influences

While this study provides a snapshot of the bacterial communities, it is crucial to consider potential anthropogenic influences. Runoffs from nearby agricultural fields, influxes from wastewater, or even recreational activities can introduce pollutants or excess nutrients, influencing the microbial community structure [76,77]. Monitoring these external influences can offer insights into the anthropogenic pressures on the pond [78].

### 4.7. Future Directions: From Observation to Manipulation

While observational studies lay the foundation, experimental manipulations can offer deeper insights. Setting up controlled microcosm or mesocosm experiments manipulating variables like nutrient levels, pollutant influx, or even biotic interactions can help decipher the drivers of bacterial community dynamics [27,34,65]. Integrating techniques like stable isotope probing or single-cell genomics could further refine our understanding, allowing us to pinpoint specific bacterial groups’ roles within the community. The Yonghwasil pond’s bacterial community dynamics offer a glimpse into the intricate interactions shaping freshwater ecosystems. While academically enriching, these insights have broader environmental implications, emphasizing the need for conservation and sustainable management of these vital habitats.

## 5. Conclusions

Yonghwasil Pond, located within the confines of the National Institute of Ecology, is a tribute to the delicate equilibrium and symbiotic coexistence in the natural world. This body of water serves as a valuable living laboratory, providing valuable knowledge on freshwater ecology, biodiversity, and the intricate interactions between aquatic and terrestrial ecosystems. The pond holds an importance that extends beyond its immediate aquatic environment. These plants, via their root systems and leaf litter, emit organic chemicals into the aquatic environment [79]. These nutrient-rich chemicals facilitate microbial communities’ proliferation and metabolic functions, particularly bacteria, inside the pond environment. Conversely, these bacterial assemblages facilitate the process of organic matter breakdown, liberating vital nutrients into the aquatic environment, which are subsequently available for absorption by plants and other species inhabiting the water. The symbiotic link between the microbiota and plants facilitates an ongoing process of nutrient exchange, enhancing both entities’ well-being and efficiency.

In addition, vegetation, including herbaceous plants and trees, along the periphery of Yonghwasil Pond serves as a natural barrier, effectively mitigating the entry of contaminants and particulate matter into the water body. The natural filtration system not only improves water quality but also creates a favorable environment for the flourishing of microbiome. Microbial communities play a crucial role in water purification by decomposing toxins and absorbing surplus nutrients, mitigating eutrophication and algal blooms. This study’s profound bacterial community dynamics enrich our academic understanding and have far-reaching implications for ecology, conservation, and public health.

### 5.1. Recapitulating the Findings

At the heart of our exploration was the revelation of the dominance of *Proteobacteria* across varied niches within the pond, underscoring their ecological versatility. The nuanced interplay between *Actinobacteria* and *Chloroflexi* further illuminated how specific bacterial groups respond to subtle environmental gradients, from aqueous to semi-solid to solid matrices. The pronounced presence of Cyanobacteria in water samples reminded us of the delicate balance of photosynthesis and nutrient dynamics, with potential ramifications for water quality and trophic interactions. The rich microbial tapestry of mud samples highlighted sediments’ often-underestimated role as reservoirs of biodiversity and hotspots of microbial interactions.

### 5.2. Implications for Freshwater Ecology

Beyond the confines of the Yonghwasil pond, these findings hold broader implications for freshwater ecology. Bacterial communities, often termed the “unseen majority”, drive pivotal biogeochemical cycles from carbon to nitrogen to sulfur. Their dynamics can influence nutrient availability, organic matter decomposition, and energy flow within the ecosystem. The observed bacterial profiles, rich in metabolic diversity, suggest a robust ecosystem capable of withstanding perturbations and ensuring sustained ecosystem functioning.

### 5.3. Conservation and Public Health Perspectives

From a conservation standpoint, understanding bacterial community dynamics is paramount. Shifts in these communities can serve as early warning signs of ecosystem health deterioration. For instance, a surge in cyanobacterial dominance could hint at eutrophic conditions, potentially stemming from nutrient runoffs or other anthropogenic influences. Such insights can guide conservation efforts, from habitat restoration to pollution control. Furthermore, certain bacterial groups, especially *Cyanobacteria*, have public health implications. Blooms of toxin-producing cyanobacterial species can jeopardize water quality, posing risks to human health. Thus, monitoring and understanding these bacterial dynamics become an ecological and public health imperative. The findings also call for technological applications. With their metabolic prowess, bacterial communities can be harnessed for biotechnological applications. The possibilities are vast and promising, from bioremediation of polluted waters to harnessing photosynthetic bacteria for bioenergy production. The rich microbial diversity observed, especially in sediment samples, could be a goldmine for novel bioactive compounds, potentially with pharmaceutical or industrial applications.

### 5.4. Charting the Future Trajectory

While this study has illuminated the microbial landscape of the Yonghwasil pond, it is merely the tip of the iceberg. The Yonghwasil pond serves as a microcosm of freshwater ecosystems’ myriad challenges and opportunities. As we stand at the crossroads of ecological challenges, from climate change to biodiversity loss, studies like this become beacons of knowledge, guiding our path forward. They remind us of the delicate balance of life, the profound interconnections, and our pivotal role as stewards of the environment. Future studies could employ metagenomic, metatranscriptomic, and metaproteomic approaches to delve deeper into these bacterial communities’ functional potentials. Additionally, long-term monitoring could unravel the temporal dynamics of these bacterial communities, offering insights into their adaptability and resilience in the face of environmental perturbations.

In summary, Yonghwasil Pond, characterized by its abundant microbial diversity shaped by the surrounding plants, is a notable ecological asset within the National Institute of Ecology. The dynamic relationship between the bacterial communities inhabiting the pond and the adjacent flora, including plants and trees, highlights the significance of conserving and conducting research on these ecosystems. As the exploration of microbial ecology progresses, habitats such as Yonghwasil Pond are expected to contribute significantly, facilitating human endeavors to establish a sustainable and mutually beneficial relationship with the natural environment.

## Figures and Tables

**Table 1:** Physio-chemical properties of soil, mud, and water samples from pond. TN stands for Total Nitrogen; TP stands for Total Phosphate; TOC stands for Total Organic Carbon; NH^4+^ stands for ammonium; NO^3−^ stands for nitrate; ND is not detected.

Sample ID	Texture	pH	TN (%)	TP (mg/kg)	TOC (%)	NH^4+^ (mg/kg)	NO^3−^(mg/kg)
L1-W	Water	4.9	0.96	0.02	8.20	0.10	0.70
L1-M	Mud	5.9	0.26	1091.29	6.42	125.74	ND
L1-T	Mud	5.7	0.12	496.01	5.24	3.12	ND
L1-Z	Mud	5.6	0.05	480.03	2.59	ND	ND
L1-R	Mud	5.7	0.43	907.58	10.68	20.19	ND
L1-B	Soil	5.6	0.35	850.21	6.70	3.91	21.32

## Data Availability

The sequencing data generated and analyzed in this study have been deposited in the NCBI Sequence Read Archive (SRA) under the BioProject accession number PRJNA1187221.

## Supplementary Materials

The following supporting information can be downloaded at: https://www.mdpi.com/article/10.3390/microorganisms12122547/s1, Figure S1: A schematic diagram showing the samples collection locations in the pond.
