# Supplementary material for: Bacterial Diversity in the Different Ecological Niches Related to the Yonghwasil Pond (Republic of Korea)
Source: Microorganisms. 2024 Dec 11;12(12):2547. doi: 10.3390/microorganisms12122547 (PMC11677111; doi:10.3390/microorganisms12122547)
Supplement: Supplementary file 1 [file microorganisms-12-02547-s001.zip › microorganisms-3313314-Figure S1.pptx]

## Slide 1
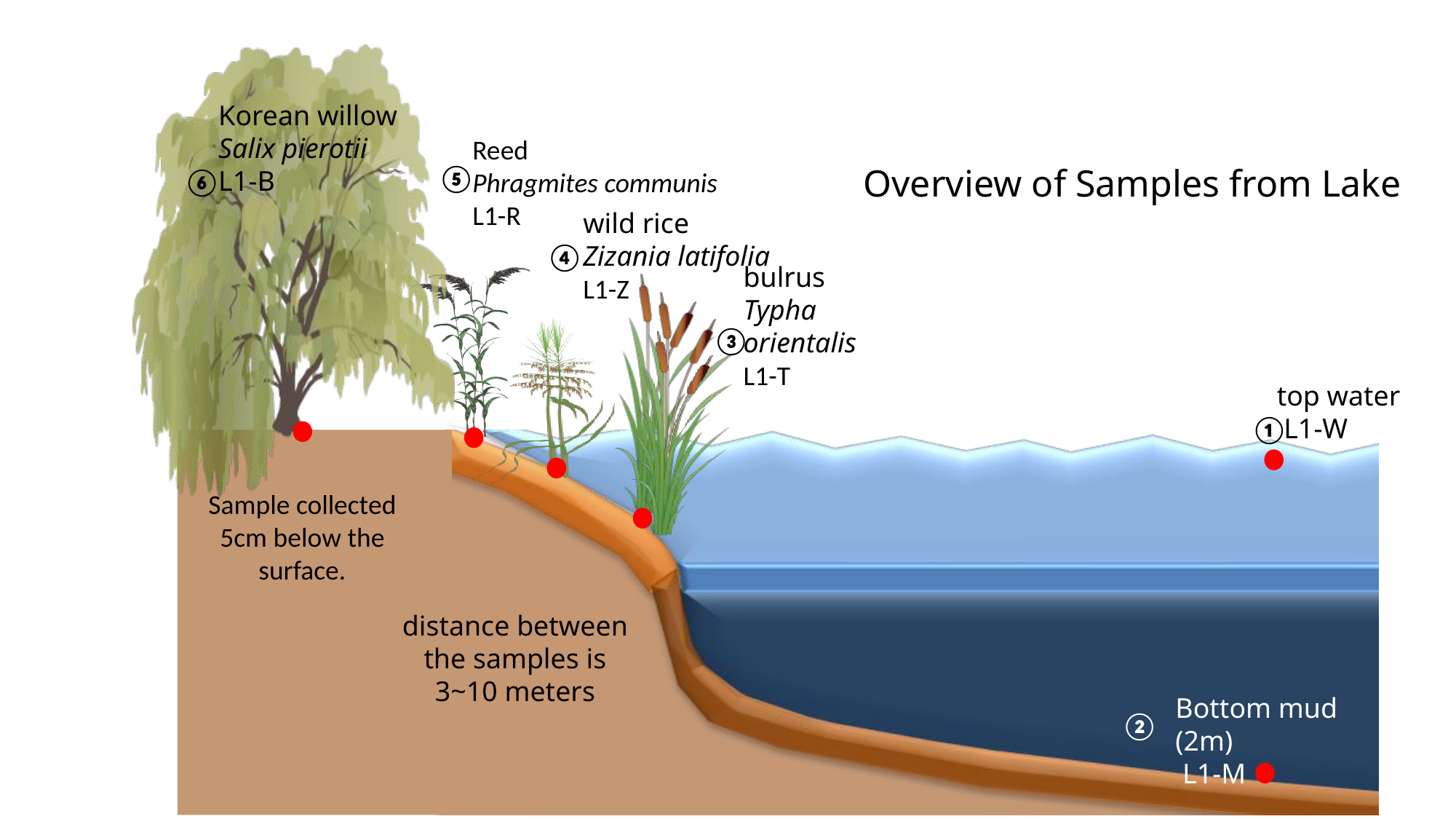

Korean willow
Salix pierotii
L1-B
⑥
Reed
Phragmites communis
L1-R
# Overview of Samples from Lake
⑤
wild rice
Zizania latifolia
L1-Z
④
bulrus
Typha orientalis
L1-T
③
top water
 L1-W
①
Sample collected 5cm below the surface.
distance between the samples is 3~10 meters
Bottom mud (2m)
 L1-M
②
